# Genetic analysis and mapping of adult plant stripe rust resistance loci in CIMMYT wheat ‘Kijil’ under Mexican and Chinese field environments

**DOI:** 10.1371/journal.pgen.1012039

**Published:** 2026-06-18

**Authors:** Shanshan Yan, Lichao Teng, Menghan Xi, Chan Yuan, Liang Wang, Shunda Li, Julio Huerta-Espino, Sridhar Bhavani, Ravi P. Singh, Caixia Lan

**Affiliations:** 1 Hubei Hongshan Laboratory, National Key Laboratory of Crop Genetic Improvement, College of Plant Science and Technology, Huazhong Agricultural University, Wuhan, Hubei, China; 2 Campo Experimental Valle de México, Instituto Nacional de Investigaciones Forestales, Agrícolas y Pecuarias (INIFAP), Chapingo, Mexico; 3 International Maize and Wheat Improvement Center (CIMMYT), Texcoco, Mexico; Pennsylvania State University, UNITED STATES OF AMERICA

## Abstract

Stripe rust, caused by *Puccinia striiformis* f. sp. *tritici*, can cause severe yield losses in wheat (*Triticum aestivum* L.) during epidemics. Breeding resistant wheat varieties remains the most cost-effective approach to manage this disease; and the identification of new resistance loci is essential for maintaining genetic diversity. The CIMMYT-derived wheat line ‘Kijil’ was highly resistant to stripe rust in both Mexican and Chinese environments. A population of 153 F₅ recombinant inbred lines (RILs) was derived from a cross between Kijil and the susceptible parent ‘Apav#1’. The population was phenotyped for stripe rust resistance across seven environments in two countries and genotyped using a genotyping-by-sequencing (GBS) platform. Inclusive composite interval mapping (ICIM) was used to construct a genetic map and identify significant resistance quantitative trait loci (QTLs) using 5,468 polymorphic markers. Mapping revealed the known resistance loci *Yr29*, *Yr30* and *QYr.hzau-3AS*, along with two novel loci, *QYr.hzau-2BS* and *QYr.hzau-5DL*, across both Chinese and Mexican rust environments. Among these, *QYr.hzau-2BS* accounted for 11.75% to 19.19% of the phenotypic variance. A corresponding KASP marker, KASP_2BS, was developed to facilitate marker-assisted selection. Based on the mapping interval, two candidate genes underlying this locus were predicted. Further analysis revealed that *Yr29* showed significant additive effects with other stripe rust resistance genes/loci, and the combination of *Yr29*, *Yr30*, and *QYr.hzau-2BS* reduced disease severity by up to 67.8%. Our findings suggest that Kijil and RILs carrying *Yr29*, *Yr30,* and *QYr.hzau-2BS* can serve as valuable donors for breeding wheat varieties with improved stripe rust resistance.

## Author summary

Stripe rust is an important disease that seriously threatens the yield and quality of wheat. It is crucial to explore new resistant resources and cultivate durable resistant varieties in the current breeding programme. In this study, we analysed the genetic basis of resistance to stripe rust in the wheat line “Kijil”, which exhibits adult plant resistance (APR) to *Puccinia striiformis* f. sp*. tritici* (*Pst*) in multiple environments, including resistance to prevalent races under Chinese and Mexician rust environments. Through genetic mapping, we identified five quantitative trait loci for stripe rust, including two new resistance loci. A closely linked KASP marker, KASP_2BS, was developed for the *QYr.hzau-2BS*, which can be used for rapid and accurate screening of resistant plants in early breeding populations. Meanwhile, within this QTL region, we screened two candidate genes based on expression analysis. In addition, pyramiding *QYr.hzau-2BS* with known APR genes, *Yr29* and *Yr30*, significantly enhanced resistance, reducing disease severity to low levels approaching near immunity. In conclusion, this study provides new genetic resources, breeding friendly molecular markers and effective gene pyramiding strategies to breed stripe rust resistant wheat. ‘Kijil’ and the lines containing resistance loci have important breeding utilization value.

## 1 Introduction

Wheat is one of the world’s most important food crops, providing a major source of energy and protein for humans [1] and accounting for about 30% of global cereal consumption (FAO, https://www.fao.org). However, wheat yields and quality are seriously threatened by rust epidemics [2–4]. Stripe, or yellow rust (YR), caused by *Puccinia striiformis* f. sp. *tritici* (*Pst*), is one of the most destructive wheat diseases worldwide. It is estimated that wheat rust causes global economic losses amounting to billions of US dollars annually [5]. The frequency and severity of rust outbreaks have increased in recent years, driven by the emergence of highly virulent races and changing climatic conditions [6]. Developing and deploying new, durable sources of resistance is therefore one of the most economical and environmentally sustainable strategies for disease management.

Resistance to YR in wheat can be broadly categorized into two types: seedling resistance or all-stage resistance (ASR), and adult plant resistance (APR). ASR, also known as vertical resistance, is typically conferred by single, major-effect genes that are expressed throughout the plant’s life cycle [7]. However, this type of resistance is often short-lived in the field due to pathogen evolution and the emergence of new virulent races [8]. Large-scale deployment of cultivars with ASR genes has repeatedly led to yield losses due to the presence of new virulent *Pst* races resulting in the breakdown of resistance genes, such as *Yr9*, *Yr10*, and *Yr24/Yr26* in China [9,10]. In contrast, APR — also referred to horizontal resistance — is controlled by multiple minor-effect genes and is usually expressed at later growth stages [11]. APR is generally considered more durable, as it tends to be non-race specific. Several well-known APR genes, *Yr18/Lr34* [12], *Yr29/Lr46* [13], *Yr46/Lr67* [14], and *Yr30/Sr2* [15], have provided effective resistance to multiple diseases for over a century. To date, 87 stripe rust resistance genes have been officially catalogued, and more than 406 QTLs associated with stripe rust resistance have been mapped to the wheat reference genome [16,17], including contributions from over 20 wheat-related species. Among these, ASR genes, *YrU1* [18], *Yr5/Yr7/YrSP* [19], *Yr9* [20], *Yr27* [21], *Yr10/YrNAM* [22], *Yr15* [23], *Yr28* [24], *Yr83* [25], and *Yr87/Lr85* [16] have been successfully cloned and most of them encoded a nucleotide-binding leucine-rich repeat (NLR) protein. Unfortunately, new virulent races were identified for them in China with exceptions of *Yr5* and *Yr15*. In contrast, three cloned APR genes, *Yr18/Lr34*, *Yr36* and *Yr46/Lr67*, operate through mechanisms distinct from typical NLR‑mediated race‑specific immunity, providing durable, non‑race‑specific resistance via metabolic or signaling pathways. For instance, *Yr18/Lr34* encodes an ABC transporter postulated to enhance cell‑wall defenses by transporting metabolites such as abscisic acid and sinapyl alcohol [12,26]. *Yr36* encodes a kinase‑START domain protein whose activity is temperature‑sensitive and regulates the homeostasis of reactive oxygen species (ROS) [27]. *Yr46/Lr67* encodes a hexose transporter that likely restricts pathogen growth by modulating sugar flux. Furthermore, the recently cloned gene, *Yr84*, derived from *Triticum dicoccoides* and located on 1BS, confers resistance through a paired NLR system — tightly linked, head‑to‑head oriented coiled-coil NLR (CNL) and NLR genes that function synergistically [28]. This represents the first reported case of wheat stripe rust resistance mediated by a cooperative NLR pair, offering a novel genetic and mechanistic paradigm for disease resistance. Given this breakthrough yet faced with the ongoing challenge of limited availability of resistance genes in cultivated wheat and the constant evolution of new pathogen virulence, the discovery and strategic combination of new resistance loci remain paramount for sustainable stripe rust management.

The CIMMYT-derived wheat line ‘Kijil’ was susceptible at the seedling stage to both Mexican and Chinese *Pst* races but showed high levels of APR under field conditions. In this study, we developed 153 F₅ recombinant inbred lines (RILs) from a cross between the susceptible cultivar ‘Apav#1’ and Kijil. The objectives of this study were to: (1) elucidate the genetic basis of APR to YR in Kijil; (2) identify new resistance loci using molecular markers under multi-environment tests and develop molecular markers associated with the newly identified loci for wheat breeding programs; and (3) assess the interaction among detected resistance loci under both Chinese and Mexican field environments.

## 2 Results

### 2.1 Phenotypic analysis

**Fig 1:**
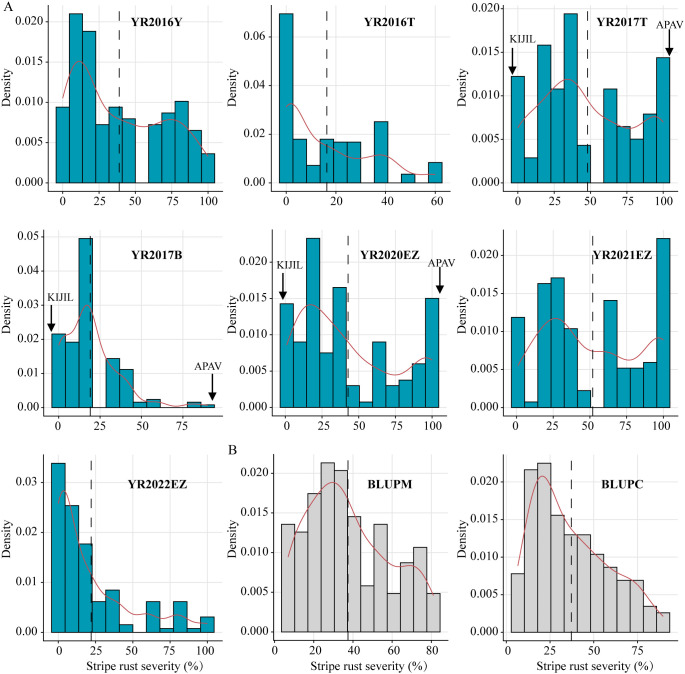
Frequency distribution of stripe rust severity in a wheat RIL population under different field environments. **(A)** Frequency distributions for final disease severity (FDS) of stripe rust for 153 ‘Apav#1’ × ‘Kijil’ F_5_ recombinant inbred lines (RILs) in field trials at Obregón, Mexico, during 2015-2016 (YR2016Y) and at Toluca, Mexico, during 2015-2016 (YR2016T) and 2016-2017 (YR2017T) and at Batan, Mexico, during 2016-2017 (YR2017B); FDS for stripe rust at Ezhou, Hubei Province, China, during 2019-2020 (YR2020EZ), 2020-2021 (YR2021EZ) and 2021-2022 (YR2022EZ). **(B)** Frequency distributions of the best linear unbiased prediction (BLUP) values for stripe rust severity. BLUPM and BLUPC: the unbiased linear predicted values of phenotypic data from the Mexican and Chinese environments, respectively. The vertical dashed black lines represent the mean disease severity for each environment.

At the seedling stage, the infection types (IT) of Apav#1 and Kijil were 7–8 and 6–7, respectively, against *Pst* races CYR33 and CYR34. At the adult plant stage, Apav#1 showed final disease severity (FDS) ranging from 40% to 100% with susceptible (S) or moderately susceptible (MS) reactions across seven test environments, whereas Kijil displayed 1MS and 1MR for FDS and host reactions, respectively, at Toluca and Batan, Mexico (Fig 1A). In Chinese YR nurseries, Kijil exhibited FDS and host reactions of 0–1R, the mean FDS of the RIL population ranged from 22.04% to 51.93% (Fig 1A and S1 Fig); in Mexican environments, the mean FDS across the RIL population ranged from 16.35% to 38.86% (Fig 1A and S1 Table). The unbiased linear predicted values of phenotypic data from the Mexican (BLUPM) and Chinese environments (BLUPC) had a mean value of 37.46% and 37.18%, respectively, and both approximately followed a normal distribution (Fig 1B). In all test environments, the frequency distribution of RILs for YR severity was continuous, indicating that APR was quantitatively inherited in this population. Mendelian segregation analysis of observed and expected frequencies in three phenotypic classes suggested the presence of two to five resistance genes with additive effects, depending on the test environment (Table 1).

**Table 1:** Estimated number of resistance genes that conferred adult plant resistance to stripe rust based on the Mendelian segregation ratios in ‘Apav#1’ × ‘Kijil’ recombinant inbred lines population.

Category	YR2016Y	YR2016T	YR2017T	YR2017B	YR2020EZ	YR2021EZ	YR2022EZ
**HTPR**	26	13	6	27	21	16	2
**HTPS**	10	5	20	22	29	30	4
**Others**	117	134	127	104	98	104	137
**Total**	153	152	153	153	148	150	143
**No. of genes**	3	4	3	2	2	2	5
***P*-value** ^**a**^	0.00*	0.81	0.02*	0.23	0.30	0.03*	0.50

HPTR, homozygous parental type resistant (RILs showing a similar phenotype as the resistant parent); HPTS, homozygous parental type susceptible (RILs showing a similar phenotype as the susceptible parent); Others, RILs showing different responses from the above two categories; YR2016Y, final stripe rust severity, Ciudad Obregon (Mexico) 2015–2016; YR2016T, Toluca (Mexico) 2016; YR2017T Toluca (Mexico) 2017; YR2017B, Batan, (Mexico) 2017; YR2020EZ, Ezhou (China) 2020; YR2021EZ, Ezhou (China) 2021; YR2022EZ, Ezhou (China) 2022.

^a^significant *P* value (*) indicates deviation from the expected ratio at *P* < 0.05.

Pearson correlation coefficients of FDS among RILs ranged from 0.67 to 0.83 in Mexican environments and 0.57 to 0.71 in Chinese environments (Table 2). In addition, FDS was significantly correlated across all test environments, with correlation coefficients ranging from 0.49 to 0.66. These positive correlations between Mexican and Chinese trials indicated that Kijil conferred broad resistance to *Pst* across both environments.

**Table 2:** Phenotypic Pearson’s correlations for stripe rust in the 153 ‘Apav#1’ × ‘Kijil’ F_5_ RIL population using the final disease severity in each environment.

Environment	YR2016T	YR2016Y	YR2017T	YR2017B	YR2020EZ	YR2021EZ
**YR2016Y**	0.70**					
**YR2017T**	0.83**	0.72**				
**YR2017B**	0.70**	0.67**	0.81**			
**YR2020EZ**	0.65**	0.59**	0.63**	0.55**		
**YR2021EZ**	0.61**	0.66**	0.61**	0.52**	0.70**	
**YR2022EZ**	0.61**	0.65**	0.54**	0.49**	0.57**	0.71**

YR2016Y, final stripe rust severity, Ciudad Obregon (Mexico) 2015-–2016; YR2016T, Toluca (Mexico) 2016; YR2017T, Toluca (Mexico) 2017; YR2017B, Batan (Mexico) 2017; YR2020EZ, Ezhou (China) 2020; YR2021EZ, Ezhou (China) 2021; YR2022EZ, Ezhou (China) 2022. ^**^ Significant at *P* < 0.01.

### 2.2 Genetic linkage maps

A total of 5,468 polymorphic markers, comprising 4,480 presence-absence variant (PAV) markers, 984 single nucleotide polymorphism (SNP) markers, 3 simple sequence repeat (SSR) markers and 1 Kompetitive Allele‑Specific PCR (KASP) marker (Table A in S1 File), were assigned to 42 linkage groups distributed across the A, B, and D genomes, with 1,826, 3,163, and 479 markers, respectively (Table B in S1 File). The genetic map spanned 8,523.94 cM, with an average marker density of 1.55 cM per marker. Linkage maps harboring identified QTLs are shown in the present study (Table C in S1 File).

### 2.3 Mapping of adult plant stripe rust resistance loci

**Fig 2:**
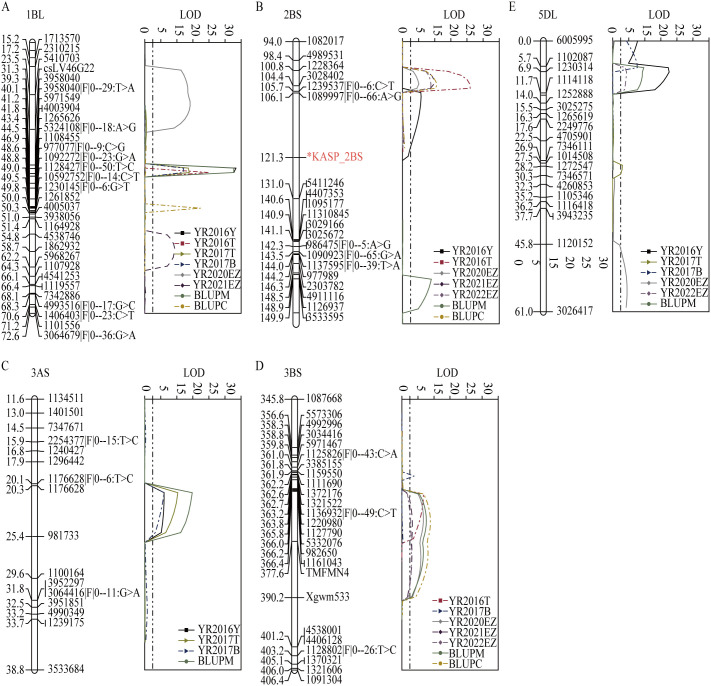
Quantitative trait loci (QTL) likelihood plots for stripe rust on five wheat chromosomes. **(A)** chromosome 1BL. **(B)** chromosome 2BS. **(C)** chromosome 3AS. **(D)** chromosome 3BS. **(E)** chromosome 5DL. The significant LOD threshold was based on 1,000 permutations. Positions in cM of the molecular markers along chromosomes are shown on the vertical axes using cumulated genetic distances. YR2016Y: phenotypic data for stripe rust resistance at Ciudad Obregon, Mexico, during 2016-2017 crop season; YR2016T, YR2017T and YR2017B: phenotypic data for stripe rust resistance at Toluca and Batan, Mexico, during 2016 and 2017 respectively; YR2020EZ, YR2021EZ, YR2022EZ: phenotypic data for stripe rust resistance at Ezhou, China, during 2019-2020, 2020-2021 and 2021-2022 crop seasons; BLUPM and BLUPC: the unbiased linear predicted values of phenotypic data from the Mexican and Chinese environments, respectively.

Five QTLs for YR resistance, *QYr.hzau-1BL*, *QYr.hzau-2BS*, *QYr.hzau-3AS*, *QYr.hzau-3BS*, and *QYr.hzau-5DL*, were detected on chromosomes 1BL, 2BS, 3AS, 3BS, and 5DL, respectively, using ICIM software based on 1,000 permutations. All QTLs were contributed by the resistant parent Kijil (Table 3). Among these, *QYr.hzau-1BL*, located on chromosome 1BL, was consistently detected. It was identified in seven individual field environments (YR2016Y, YR2016T, YR2017T, YR2017B, YR2020EZ, YR2021EZ, YR2022EZ) and also mapped in the composite BLUP values for both Mexico (BLUPM) and China (BLUPC) (Table D in S1 File), jointly explaining 16.03–33.71% of the phenotypic variation (Table 3; Fig 2A). Based on the physical positions of flanking markers, this QTL likely corresponds to the known resistance gene *Yr29*. *QYr.hzau-2BS* was detected in five individual field environments (YR2016Y, YR2016T, YR2020EZ, YR2021EZ, and YR2022EZ) as well as in the composite BLUP analyses for both Mexico (BLUPM) and China (BLUPC) (Table E in S1 File), jointly explaining 5.03–23.39% of the phenotypic variation. It was closely linked to markers 1228364 and 3021343 (Table 3; Fig 2B) and showed no linkage to the known stripe rust resistance gene *Yr27*. *QYr.hzau-3AS*, flanked by markers 1176628 and 981733 (Table F in S1 File), explained 5.19–17.53% of the variation and was only detected in Mexican environments (Table 3; Fig 2C). *QYr.hzau-3BS,* located on chromosome 3BS between markers 1321522 and *Xgwm533*, was detected in five field environments (YR2016T, YR2017B, YR2020EZ, YR2021EZ, and YR2022EZ) (Table G in S1 File). Its effect was further supported by its association with the BLUPM and BLUPC, explaining 3.48–12.77% of the variation (Table 3; Fig 2D). Based on its chromosomal location, it likely corresponds to *Yr30*. *QYr.hzau-5DL* was detected in three Mexican environments and two Chinese environments between markers 1102087 and 3026417 (Table H in S1 File), explaining 4.15–17.40% of the phenotypic variation (Table 3; Fig 2E).

**Table 3:** Position and effect of quantitative trait loci (QTL) detected for adult plant resistance (APR) to stripe rust (YR) in both Mexican and Chinese rust environment, using final disease severity and best linear unbiased prediction (BLUP; BLUPM for Mexico, BLUPC for China) of 153 RILs derived from the cross of ‘Apav#1’ × ‘Kijil’.

QTL	TraitName	Position	LeftMarker	RightMarker	LOD	PVE(%)	Add^a^
***QYr.hzau-1BL***	YR2020EZ	26	*5410703*	*csLV46G22*	14.12	24.21	-17.77
	YR2016Y	40	*3958040*	*3958040|F|0--29:T > A*	28.40	33.71	-19.05
	YR2017B	40	*3958040*	*3958040|F|0--29:T > A*	12.12	16.08	-7.09
	BLUPM	40	*3958040*	*3958040|F|0--29:T > A*	27.71	24.88	-11.25
	YR2016T	41	*3958040|F|0--29:T > A*	*5971549*	19.96	21.45	-8.34
	YR2017T	41	*3958040|F|0--29:T > A*	*5971549*	13.84	23.54	-14.75
	BLUPC	49	*1092272|F|0--23:G > A*	*1128427|F|0--50:T > C*	17.29	24.07	-12.20
	YR2021EZ	58	*4538746*	*1862932*	9.29	16.03	-15.02
***QYr.hzau-2BS***	YR2021EZ	103	*1228364*	*3028402*	9.75	17.44	-16.98
	YR2020EZ	104	*1228364*	*3028402*	5.06	7.08	-12.05
	YR2022EZ	104	*1228364*	*3028402*	8.82	9.25	-10.46
	BLUPC	104	*1228364*	*3028402*	10.72	13.71	-11.62
	YR2016T	105	*3028402*	*1239537|F|0--6:C > T*	20.98	23.39	-5.20
	YR2016Y	107	*1089997|F|0--66:A > G*	*KASP_2BS*	5.77	5.03	-6.75
	BLUPM	150	*2317427*	*3021343*	8.93	5.89	-5.84
***QYr.hzau-3AS***	YR2016Y	21	*1176628*	*981733*	6.05	5.19	-7.41
	YR2017T	21	*1176628*	*981733*	10.31	17.53	-12.64
	YR2017B	21	*1176628*	*981733*	6.08	7.63	-4.84
	BLUPM	21	*1176628*	*981733*	14.81	10.97	-7.40
***QYr.hzau-3BS***	YR2017B	363	*1321522*	*1136932|F|0--49:C > T*	3.77	4.42	-3.69
	YR2016T	367	*3029878*	*TMFMN4*	6.75	6.05	-4.40
	YR2020EZ	372	*3029878*	*TMFMN4*	6.70	11.31	-12.11
	BLUPM	373	*3029878*	*TMFMN4*	7.61	5.93	-5.44
	BLUPC	373	*3029878*	*TMFMN4*	8.90	12.77	-8.85
	YR2021EZ	374	*3029878*	*TMFMN4*	3.27	6.01	-9.20
	YR2022EZ	382	*TMFMN4*	*Xgwm533*	3.03	3.48	-6.47
***QYr.hzau-5DL***	YR2017B	6	*1102087*	*1230314*	7.67	9.58	-5.43
	BLUPM	6	*1102087*	*1230314*	9.68	6.39	-5.66
	YR2016Y	7	*1230314*	*1114118*	17.58	17.40	-13.57
	YR2022EZ	7	*1230314*	*1114118*	4.32	4.15	-7.07
	YR2017T	28	*1014508*	*1272547*	3.02	4.36	-6.37
	YR2020EZ	56	*1120152*	*3026417*	4.48	8.08	-10.43

LOD, logarithm of odds score of QTL peak; PVE, proportion of phenotypic variance explained by the QTL; Add, additive effect of phenotypic for each QTL.

^a^negative values indicate resistance alleles derived from Kijil.

### 2.4 Development and validation of KASP molecular marker for *QYr.hzau-2BS*

**Fig 3:**
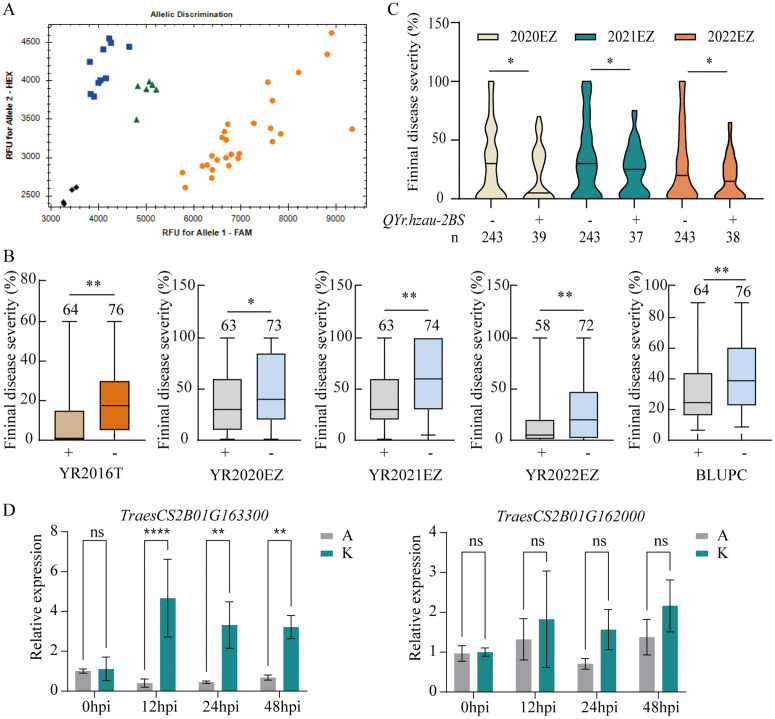
Development of a KASP marker and functional characterization of candidate genes for *QYr.hzau-2BS.* **(A)** KASP genotyping for *QYr.hzau-2BS*. Blue, orange, and green dots represent susceptible, resistant and heterozygous genotypes, respectively; black dots indicate no template control. **(B)** Association analysis for *QYr.hzau-2BS* using the KASP marker with stripe rust final disease severity across five environments (YR2016T, YR2020EZ, YR2021EZ, YR2022EZ and BLUPC). “−” indicates the absence of the QTL locus, whereas “+” indicates its presence. Significance levels are denoted by asterisks (^*^, *P* < 0.05; ^**^, *P* < 0.01; ns = non-significant). The sample size was designed. **(C)** Effect of *QYr.hzau-2BS* on stripe rust severity in a panel of 416 wheat accessions. Violin plots show the distribution of final disease severity (%) among 416 wheat accessions, grouped by the absence (−) or presence (+) of the *QYr.hzau-2BS* resistance allele. Field trials were conducted in Ezhou, China, during the 2020 (2020EZ), 2021 (2021EZ), and 2022 (2022EZ) seasons. The thick line inside each violin represents the median. Sample sizes for each group are labeled. Asterisks denote significant differences between groups within a year (*P* < 0.05, *t*-test). **(D)** Relative expression patterns of *TraesCS2B01G162000* and *TraesCS2B01G163300* in Apav#1 and Kijil at 0, 12, 24, and 48 hours post-inoculation (hpi) with a mixture of *Puccinia striiformis* f. sp. *tritici* races CYR33 and CYR34. A, represents susceptible parent Apav#1; K, represents resistant parent Kijil. Error bars represent standard error of the mean (SEM) of three biological replicates. Statistically significant differences between genotypes at each time point are indicated (*, *P* < 0.05; **, *P* < 0.01; ns, not significant).

Considering the stable phenotypic effect of *QYr.hzau-2BS* across multiple environments, we developed a KASP marker based on the closely linked SNP marker 1239537|F|0--6:C > T and it was named KASP_2BS (S2 Table). Genotyping results showed clustering of the alleles into two distinct groups (Fig 3A). Linkage analysis confirmed that KASP_2BS is located at the peak of the QTL and it was placed to the physical position of 134.55 Mb on chromosome arm 2BS based on the Chinese Spring RefSeq v1.0 genome. We used this marker to genotype the entire RIL population, and it was significantly associated with disease severity reductions in one Mexican environment (YR2016T) and three Chinese field environments (YR2020EZ, YR2021EZ, YR2022EZ, and BLUPC) (Table I in S1 File), with reductions of 8.89%, 12.61%, 18.13%, 13.64%, and 11.72%, respectively (Fig 3B). Furthermore, this marker also showed disease severity reductions of 3.00%, 5.13%, 4.92%, and 5.19%, respectively, under YR2016Y, YR2017T, YR2017B, and BLUPM rust environments (S2 Fig, S3 Table). To further validate the phenotypic effect and breeding value of this locus in diverse genetic backgrounds, we genotyped 416 wheat accessions with KASP_2BS and identified 38 lines carrying the resistance allele. Marker–trait association analysis within this population demonstrated that the resistance allele was significantly associated with low stripe rust severity (*P* < 0.05) across all three tested environments (2020EZ, 2021EZ, and 2022EZ) with average reductions in disease severity of 11.66%, 10.22%, and 12.48% in the 2020EZ, 2021EZ, and 2022EZ environments, respectively (Fig 3C) compared to lines carrying the susceptible allele (Table J in S1 File).

Based on linkage marker analysis and RNA-seq of two parents in the adult plant stage, 19 annotated genes were identified within the target chromosomal region, of which three genes showed expression (FPKM > 1) after inoculation of stripe rust at the adult plant stage (S4 Table). Integrated functional annotation revealed that two candidate genes potentially associated with plant disease resistance, such as *TraesCS2B01G162000* encoding an E3 ubiquitin ligase and *TraesCS2B01G163300* belonging to the multidrug and toxic compound extrusion (MATE) family.

We assessed the expression dynamics of the candidate genes in the resistant and susceptible parents post-inoculation by employing qRT-PCR analysis (Table K in S1 File). The results showed that *TraesCS2B01G163300* exhibited sustained upregulation in the resistant parent Kijil and the expression levels were significantly higher than those in the susceptible parent Apav#1 at 12, 24, and 48 h post-inoculation, respectively (Fig 3D). In contrast, although no statistically significant difference was observed for *TraesCS2B01G162000* between resistant and susceptible materials, its expression remained consistently higher in Kijil than in Apav#1 (Fig 3D). Therefore, *TraesCS2B01G163300* was identified as a core candidate gene that is strongly induced upon pathogen infection, while *TraesCS2B01G162000* represents a potential candidate gene with a consistent expression trend in the resistant background, requiring further functional validation.

### 2.5 Interaction effects of resistance loci

**Fig 4:**
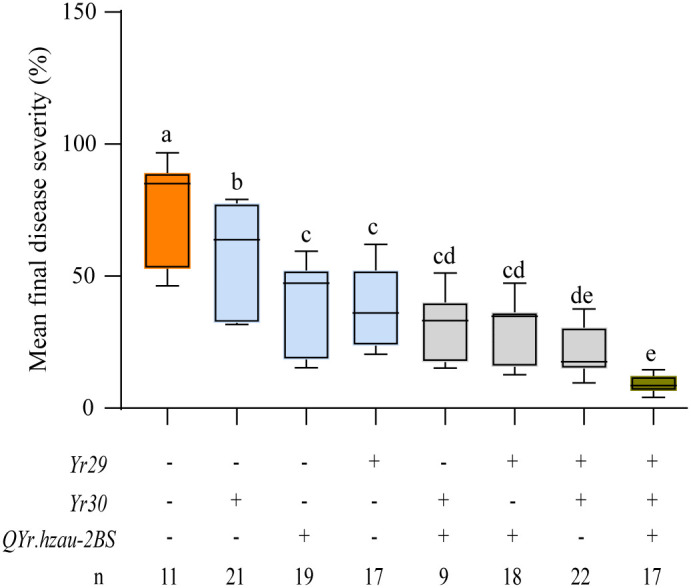
Effects of stacking resistance genes on stripe rust severity in ‘Apav#1’ × ‘Kijil’ RIL population. Box-and-whisker plots show the distribution of mean final disease severity among lines with different combinations of *Yr29*, *Yr30*, *QYr.hzau-2BS*. “−” indicates the absence of the QTL locus; whereas “+” indicates its presence. The center line, box, and whiskers showing the median, interquartile range and minimum to maximum values, respectively. One-way analysis of variance revealed significant differences (*F* [7,48] = 7.121, *P* = 7.79 × 10^-6^). Groups annotated with different lowercase letters are significantly different at *P* < 0.05 (Duncan's multiple range test). Different colors represent distinct genotype categories (no resistance genes, orange; one resistance gene, light blue; two resistance genes, gray; three resistance genes, yellow); “n” indicates the sample size of each combination.

The 153 F₅ RILs were classified into eight genotypic groups based on the molecular marker *csLV46G22*, *Xgwm533*, KASP_2BS for *Yr29*, *Yr30*, and *QYr.hzau-2BS*, respectively (Fig 4; S5 Table). Analysis of the combined data showed that RILs carrying single resistance locus (*Yr29*, *Yr30*, and *QYr.hzau-2BS*) displayed significantly lower FDS compared to the lines lacking all resistance genes. *Yr29* was the most effective, conferring the lowest mean FDS of 38.20% (Fig 4; S5 Table). The line carrying *QYr.hzau-2BS* had the mean FDS of 38.66%, whereas *Yr30* alone provided only a minor, but significant reduction (19.79%) on stripe rust. Among the two-gene combinations, *Yr29* + *Yr30*, *Yr29* + *QYr.hzau-2BS* and *Yr30* + *QYr.hzau-2BS* exhibited the mean FDS of 21.60%, 28.53% and 30.90% respectively. Lines carrying these three resistance genes showed significantly lower mean FDS than those carrying two resistance loci. The combination *Yr29 + Yr30 + QYr.hzau-2BS* exhibited the lowest mean FDS (9.15%), approaching a near-immune state (Fig 4; S5 Table).

Epistatic interactions among the resistance loci were analyzed to determine the genetic basis of the observed pyramiding effects. Linear model analysis revealed significant negative epistasis between *Yr29* and *QYr.hzau-2BS* for BLUPM, with an interaction effect of +23.08% (Table 4), indicating that *Yr29* may attenuate the resistance contribution of *QYr.hzau-2BS*. No significant pairwise interaction was detected between *Yr30* and *QYr.hzau-2BS*, nor was the three-way interaction significant. To quantify this context-dependent effect, we performed stratified linear regression analysis and found that *QYr.hzau-2BS* significantly reduced disease severity by 16.06% for BLUPM in the absence of *Yr29*, while its effect was diminished to a non-significant 1.20% when *Yr29* present in the background (S6 Table). A similar attenuating trend was also observed for BLUPC. Notably, despite the lack of a significant three-way interaction in the model, the RILs carrying all three resistance loci exhibited a near-immune phenotype. This suggests that *Yr30* may contribute to a high level of resistance through its additive effect or within a more complex polygenic network.

**Table 4:** Linear model estimates of additive and epistatic effects for *Yr29*, *Yr30*, and *QYr.hzau-2BS* on stripe rust severity in BLUPM and BLUPC environments.

Phenotype	Term	Estimate ± Std. Error (%) ^a^	p value	Significance ^b^
**BLUPM**	*Intercept*	61.21 ± 5.03	1.95E-23	***
	*Yr29*	-28.50 ± 6.36	1.56E-05	***
	*Yr30*	-7.32 ± 6.25	2.44E-01	ns
	*QYr.hzau-2BS*	-21.20 ± 6.42	1.23E-03	**
	*Yr29 × Yr30*	1.05 ± 8.24	8.99E-01	ns
	*Yr29 × QYr.hzau-2BS*	23.08 ± 8.56	7.90E-03	**
	*Yr30 × QYr.hzau-2BS*	8.47 ± 9.24	3.61E-01	ns
	*Yr29 × Yr30 × QYr.hzau-2BS*	-14.60 ± 12.13	2.31E-01	ns
**BLUPC**	*Intercept*	58.07 ± 5.02	7.07E-22	***
	*Yr29*	-19.15 ± 6.35	3.08E-03	**
	*Yr30*	-2.61 ± 6.24	6.76E-01	ns
	*QYr.hzau-2BS*	-16.68 ± 6.42	1.03E-02	*
	*Yr29 × Yr30*	-8.93 ± 8.29	2.80E-01	ns
	*Yr29 × QYr.hzau-2BS*	5.70 ± 8.55	5.06E-01	ns
	*Yr30 × QYr.hzau-2BS*	-7.30 ± 9.23	4.30E-01	ns
	*Yr29 × Yr30 × QYr.hzau-2BS*	9.51 ± 12.12	4.34E-01	ns

BLUPM and BLUPC are best linear unbiased predictions for Mexican and Chinese environments, respectively.

^a^‘Estimate’ for the main effect indicates the average change in disease severity (%) when the genotype at that locus changes from the susceptible to the resistant allele. A significant positive estimate for an interaction term indicates antagonistic epistasis.

^b^ Significance levels: ***, *P* < 0.001; **, *P* < 0.01; *, *P* < 0.05; ns, not significant.

## 3 Discussion

Developing wheat varieties with durable resistance remains a major objective of modern wheat breeding programs. In this study, Kijil, a CIMMYT-derived wheat line with superior agronomic performance, exhibited a high level of resistance to stripe rust across both Mexican and Chinese field environments. A total of five stable QTLs for APR to stripe rust were identified from Kijil, among these, the known resistance genes *Yr29* and *Yr30* were also confirmed based on the genotype and phenotype effect.

### 3.1 *QYr.hzau-1BL* (*Yr29*)

*QYr.hzau-1BL* was located in the interval between DArT markers 5410703 and 1862932, explaining 16.03–33.71% of the phenotypic variation. It was mapped to 671.27–676.07 Mb near the distal end of chromosome 1BL based on the Chinese Spring IWGSC RefSeq v1.0 (IWGSC 2018), where the well-known adult plant resistance gene *Lr46/Yr29/Sr58/Pm39/Ltn2* was closely linked to molecular marker *csLV46G22* (E. Lagudah, personal communication) and has been reported on this chromosome region within 2.6 Mb from *QYr.hzau-1BL* [29–31]. *Yr29* has been deployed globally for over six decades and is known to provide partial APR to multiple diseases, including leaf rust and stripe rust. Its effect varies among genetic backgrounds, explaining 15.1–22.1% of the YR variation in ‘Pastor’ and 11.7–43.6% in ‘Francolin #1’ [31,32]. Similarly, *QYr.hzau-1BL* reduced YR severity by 16% and 21% in the ‘Borlaug 100’ [33] and Kijil backgrounds, respectively. Collectively, these findings indicate that *QYr.hzau-1BL* corresponds to the *Yr29* locus. The results confirmed that *Yr29* contributes stable, partial resistance across diverse environments and genetic backgrounds.

### 3.2 *QYr.hzau-2BS*

*QYr.hzau-2BS* was significantly associated with stripe rust resistance under Ciudad Obregón (Mexico) and Ezhou (China) rust environments, mapping between 134.55 Mb and 135.79 Mb on chromosome 2B (IWGSC RefSeq v1.0). Several stripe rust resistance genes have been mapped on 2BS chromosome, including *Yr5/Yr7/YrSp*, *Yr27*, *Yr31*, *Yr41*, *Yr43*, *Yr53*, and *Yr72*. *Yr27* originally derived from ‘Selkirk’ and it was closely linked with the leaf rust resistance gene *Lr13* [34], which is common in CIMMYT germplasm [35].

Recently, *Yr27* was cloned from a South African cultivar ‘Kariega’ by using a high-quality genome assembly method [21]. The gene spans 23.2 kb and encodes a nucleotide-binding leucine-rich repeat (NLR) protein of 1,072 amino acids, sharing 97.3% sequence identity with *Lr13*. However, virulence to *Yr27* is now widespread, for example, the majority of Chinese races CYR32 and CYR33 as well as US races *PST*v-37 and *PST*v-52 were virulent to this gene and the virulence *Pst* race for *Yr27* was about 69.9% of 61 Chinese *Pst* isolates [36–38]. Despite this, *QYr.hzau-2BS* was consistently detected in Ezhou across multiple years in the present study, reducing mean disease severity by 12.61-18.13%. It was mapped 23.13 Mb away from *Yr27* and Kijil also lacked the gene-based molecular marker of *Yr27*. Thus, *QYr.hzau-2BS* should be a new stripe rust resistance locus from Kijil.

The KASP_2BS marker, developed for *QYr.hzau-2BS*, consistently differentiated between resistant and susceptible phenotypes across multiple environments. It was further validated in a diverse panel of 416 wheat accessions, in which the resistance allele was consistently associated with lower stripe rust severity and reduced the FDS of 10.22% to 12.48% over three field seasons. These results confirm the stability of the *QYr.hzau-2BS* effect in diverse genetic backgrounds and demonstrate the practical value of the KASP_2BS marker for both germplasm screening and marker-assisted breeding.

Through transcriptomic analysis of inoculated parents, we identified two candidate genes within the *QYr.hzau-2BS* interval that were expressed (FPKM > 1) and had resistance-related annotations, pointing to distinct mechanisms for this QTL-mediated resistance. *TraesCS2B01G162000* encodes an E3 ubiquitin ligase, a class of enzymes that play pivotal regulatory roles in plant immunity by mediating the ubiquitination and degradation of key signaling components resulting in transducing defense responses [39,40]. Wheat E3 ubiquitin ligases, such as *TaVDIP1* and *TaGW2*, suppress plant defense by degrading positive regulatory proteins [41,42]; conversely, *RFEL1* confers broad-spectrum resistance by degrading the negative regulator *TaNPR3* to stabilize *TaNPR1* and activate defense genes [39]. This reveals the dual regulatory function of E3 ligases within the disease resistance network. The expression of *TraesCS2B01G162000* in the resistant parent Kijil suggests it may positively regulate stripe rust resistance via ubiquitination.

The other candidate gene, *TraesCS2B01G163300*, belongs to the Multidrug and Toxic Compound Extrusion (MATE) transporter family. MATE proteins are primarily involved in the efflux of secondary metabolites, such as antimicrobial alkaloids or phenolics, and constitute a key component of chemical defense. In *Arabidopsis thaliana*, for instance, they precisely regulate resistance by directly controlling the transport of core defense signaling molecules such as salicylic acid [43]. Alternatively, they may have effect on the expression of defense-related genes. In rice, overexpression of *OsMATE1/2* suppresses the expression of *PR1*, *PR5* and other genes, thereby exerting a potent negative regulatory effect on disease resistance [44]. *TraesCS2B01G163300* also showed a consistent upregulation trend in the resistant background.

Although the functional annotation and expression analysis showed the role of two potential candidate genes *TraesCS2B01G162000* and *TraesCS2B01G163300* for stripe rust resistance, while their function still need more work to confirm in future, such as genetic transformation, gene editing, and protein interactome analysis of these two genes in the Kijil background or its near-isogenic lines.

### 3.3 *QYr.hzau-3BS* (*Yr30*)

Chromosome 3BS is known as a hotspot for disease resistance genes, harboring *Fhb1* [45], *Yrns-B1* (*Yr30*) [8,46], *Sr2* [47], *Lr27* [48] and *Pm70* [49]. In this study, *QYr.hzau-3BS* mapped between DArT marker 1321522 and SSR marker *Xgwm533*, explaining 3.48–12.77% of phenotypic variation. Several APR stripe rust QTL on 3BS have been reported in varieties such as ‘Opata’ [8], ‘Kukri’ [50], and ‘Renan’ [51,52]. The closely linked molecular marker *Xgwm533* was also significantly associated with resistance in Apav#1/Kijil RIL population, suggesting that *QYr.hzau-3BS* corresponds to the *Yr30* region. Although their physical position overlapped, the additional validation—such as allelism tests or fine mapping—will be required to confirm their relationship.

### 3.4 *QYr.hzau-3AS*

*QYr.hzau-3AS* represents another APR locus detected exclusively in the Mexican test environments. Only a few YR resistance genes have been reported on chromosome 3AS, such as *YrTr1*, derived from the variety ‘Tres’, conferring ASR [53,54]. *Yr76* also provides ASR distinct from ‘Tyee’ and it was flanked by SSR markers *wmc11* and *wmc532* [55]. Lillemo et al. [56] identified an APR QTL on 3AS from ‘Saar’, and it was flanked by markers *Xstm844tcac* and *Xbarc310*, and this locus was only detected in Toluca stripe rust environment. It was mapped to 7.14 Mb on chromosome 3AS based on the Chinese Spring IWGSC RefSeq v1.0 (IWGSC 2018). Despite the 13 Mb difference in the physical location between *QYr.hzau-3AS* (19.32-20.23 Mb) and QTL mapped by Lillemo et al (2008), the two loci may be the same because both were detected only in Toluca, suggesting that this locus may confer race-specific or environment-specific resistance in the adult plant stage. However, the subsequent fine-tuning tools such as high-resolution genetic mapping, allelism tests, or haplotype analysis are still needed to confirm their relationship in future.

### 3.5 QYr.hzau-5DL

To date, no named YR resistance gene has been reported on chromosome 5DL, although several resistance QTL have been mapped. Suenaga et al. [52] identified an APR locus from ‘Oligoculm’ with flanking molecular marker of *Xwmc215* (472.37 Mb) on this chromosome, while Imtiaz et al. [57] and Liu et al. [58] mapped *QYr.nsw-5DL* and *QYr.caas-5DL* near 408.66 Mb. Another QTL, *QYr.GTM-5DL*, was reported in the Chinese landrace ‘Guangtoumai’ and it was flanked by *dCAPS-5722* at 449.29–451.17 Mb [59]. In contrast, *QYr.hzau-5DL*, identified in this study, was located at 337.69–361.65 Mb within the physical interval distinct from all previously reported loci. Although it explained a relatively small effect on stripe rust (3.75–10.97%), the QTL was consistently detected across Mexican and Chinese rust environment, indicating a stable and novel APR locus to YR.

### 3.6 Additive effects of resistance loci on stripe rust

Analysis of disease severities of RILs carrying combinations of resistance loci revealed that pyramiding multiple resistance genes is an effective strategy for enhancing resistance, which is consistent with previous reports [60]. In the study, the resistance gene *Yr29* not only significantly reduced stripe rust severity but also displayed a strong additive effect when combined with other resistance loci resulting in the improvement of diesese resistance. This conclusion is supported by several studies. For example, combining *Yr29* with *QYr.nwafu-4BL.3* reduced disease severity by 16.8–27.7% [61]. In the Chinese wheat cultivar Jimai 44, carrying the combination of *Yr29* and *YrJ44*, the mean FDS was reduced to 29.5% [62]. Similarly, pyramiding *Yr29* with *Yr30* in variety Borlaug 100 reduced disease severity by 55% [33]. Through epistasis analysis of the interaction between *Yr29* and *QYr.hzau-2BS*, we detected a significant antagonistic epistasis in the BLUPM, contrasting with the typically reported additive effects of *Yr29* in combination with other resistance genes [33,61]. This antagonistic epistasis indicates that even though pyramiding *Yr29* and *QYr.hzau-2BS* results in a phenotypically effective resistance level superior to either locus alone, their combined effect is sub-additive. This provides a new genetic explanation for the performance differences of *Yr29* across different genetic backgrounds. Introducing *QYr.hzau-2BS* into a background lacking *Yr29* may represent a superior strategy for realizing the full resistance potential of *QYr.hzau-2BS*.

Although *Yr30* alone conferred a little detectable resistance in our population, it contributed to a pronounced synergistic effect in the three-gene combination with *Yr29* and *QYr.hzau-2BS*. The two-loci combination (*Yr29* + *QYr.hzau-2BS*) achieved the mean FDS of 28.53%, whereas the triple loci combination (*Yr29* + *Yr30* + *QYr.hzau-2BS*) further reduced the mean FDS to 9.15%, a level approaching immunity. This indicates that *Yr30*, despite its weak individual effect, is a key component within a polygenic resistance network, where it enhances the effects of other genes. This mode of action is similar to that of the well-known resistance gene *Lr34*, which significantly enhances overall resistance levels primarily through synergistic rather than independent action with other genes [12]. This is also consistent with the previously proposed understanding that the expression of adult plant resistance depends on the genetic background and polygenic interaction networks [63]. Consequently, the evaluation and utilization of resistance genes must transcend their individual effects and be systematically considered within specific polygenic interaction networks [64]. This provides crucial genetic insights for modern breeding strategies that aim to achieve durable resistance through genomic design and prediction, and the precise assembly of synergistic gene modules [65].

In summary, we identified five stripe rust adult plant resistance (APR) loci in the Apav#1 × Kijil population. Two of them corresponded to the known genes *Yr29* and *Yr30*, whereas *QYr.hzau-2BS* and *QYr.hzau-5DL* are likely novel loci. A total of two potential candidate genes were predicted for the stable and major-effect resistance locus *QYr.hzau-2BS* and a KASP marker was developed for this locus to facilitate its utilization in wheat breeding. In addition, we also found the significant additive effects between detected resistance loci, especially for the gene combinations of *Yr29*. For example, the combination of *Yr29*, *Yr30*, and *QYr.hzau-2BS* formed a high-resistance core module. Therefore, it will be good if wheat breeders can use the related closely linked molecular markers to pyramid this combination with other resistance loci for marker assisted selection, providing an effective strategy for developing wheat cultivars with enhanced and durable stripe rust resistance.

The genetic effects and interactions reported here were characterized within a single biparental population. Therefore, the generalizability of the novel loci and their interaction patterns across a wider genetic background need to be validated further. The complete genetic architecture of adult plant resistance is likely more complex than the network revealed in this work. The phenotypic variation observed in this study, as in all field-based analyses, resulted from the combination of genetic effects and environmental fluctuations. While we have focused on dissecting the genetic components, the influence of environmental factors on disease pressure across seasons and locations is acknowledged. Moreover, the stability of the observed epistatic interactions across diverse environments requires systematic assessment with multi-year, multi-location trials.

## 4 Materials and methods

### 4.1 Plant materials

The CIMMYT wheat line ‘Apav#1’ (CIMMYT GID 1854090; pedigree: Avocet-*YrA*/Pavon) was susceptible to stripe rust at both seedling and adult plant stages, whereas Kijil (CIMMYT GID 6342979; pedigree: Klein Don Enrique*2/3/Fret2/Weebill1//Tacupeto F2001) was susceptible at the seedling stage but showed field resistance to the same *Pst* races at the adult plant stage. A population of 153 F₅ recombinant inbred lines (RILs) was developed from the cross between Apav#1 and Kijil at CIMMYT, using the method described by Yuan et al [66]. For field screening experiments, the Chinese wheat line ‘SY-You’ was used as susceptible control and spreader line in China, while in Mexico, a mixture of Morocco and six susceptible lines derived from an ‘Avocet*’ × ‘*Attila*’* cross served as spreaders to ensure sufficient inoculum for phenotyping the RIL population. A global collection of 416 wheat accessions was assembled, including 292 cultivars, 105 landraces and 19 synthetic wheat lines. Geographically, the collection represented 242 accessions from Asia, 22 from North America, 17 from South America, 28 from Europe, 12 from Oceania, 14 from Africa, and 81 introductions from CIMMYT (Table L in S1 File).

### 4.2 Field trials and disease evaluation

The 153 RILs and their parents were evaluated for seedling resistance to YR in a greenhouse at Huazhong Agricultural University, China, using *Pst* races CYR33 and CYR34. Infection types (IT) were scored 12–14 days after inoculation based on a 0–9 scale [67]. The same materials, along with susceptible checks, were evaluated for adult plant resistance in multiple environments: Ciudad Obregón during 2015-2016 (YR2016Y), Toluca during 2016 and 2017 (YR2016T and YR2017T), El Batán in 2017 (YR2017B), and Ezhou City, Hubei Province, China, during 2019-2020, 2020-2021, and 2021-2022 (YR2020EZ, YR2021EZ, and YR2022EZ, respectively). Field experiments followed a randomized complete block design. In China, each line was sown in 1.5 m rows spaced 30 cm apart, with approximately 50 seeds per row, whereas in Mexico sowing was done as paired rows of 0.7 m length, 20 cm apart, on top of 80 cm wide raised beds. Forty RILs were randomly selected for two replications using an augmented design. A susceptible spreader row was planted alongside each plot. These 416 wheat accessions were planted and phenotyped for stripe rust severity under field conditions in Ezhou, China, during the 2020, 2021, and 2022 crop seasons. The experimental design and disease assessment followed the same protocols as described for the RIL population. In Toluca, Mexico, spreaders were inoculated at the jointing stage with *Pst* isolate Mex14.141, characterized by avirulence/virulence to *Yr1, 4, 5, 10, 15, 17, 24* and virulence to *Yr2, 3, 6, 7, 8, 9, 27, 31*, and *A* [68]. Natural infections incited epidemics at Obregon and El Batan in Mexico. In China, inoculations were conducted with a mixture of races CYR33 (avirulence/virulence formula based on seedling phenotypes: *Yr5, 8, 10, 15, 18, 24, 26, 27, 29, 32, Tr1/1, 6, 7, 9, 17, 28, 31, 43, 44, Exp2, SP*) [69] and CYR34 (*Yr1, 3, 4, H46, 5, 6, 15, 17, 18, 29, 32, SP, Sd/2, 8, 9, 10, 12, 2*4[= 26]*, 31, Su*) [70]. Inoculum was applied as a suspension of urediniospores in Soltrol 170 oil. YR disease severity was recorded using the modified Cobb’s Scale when susceptible checks reached ~80% severity [71]. A second assessment was made one week later, when the checks reached 100% severity; these values were used as final disease severity for analysis. FDS was recorded once at El Batán (YR2017B); twice at Ezhou during 2019–2021 (YR2020EZ, YR2021EZ) and once in 2022 (YR2022EZ); three times at Toluca during 2016–2017 (YR2016T) and once in 2015–2016 (YR2017T); and once at Ciudad Obregón (YR2016Y). Host response to infection was evaluated according to Roelfs et al. [72] following: R (resistant): small sized uredinia with necrosis; MR (moderately resistant): small to intermediate sized uredinia with limited sporulation and visible chlorosis/necrosis; M (moderately resistant-moderately susceptible): medium sized uredinia with moderate sporulation and some chlorosis/necrosis; MS (moderately susceptible): medium sized uredinia with abundant sporulation and no chlorosis/necrosis; and S (susceptible): large uredinia with abundant sporulation and no chlorosis/necrosis.

Phenotypic data from multiple environments were combined using Best Linear Unbiased Prediction (BLUP), calculated as:


$$\begin{array}{c} BLUP=\mu +\sum f_{i}+\sum e_{i}+\varepsilon_{i} \end{array}$$ (1)

where μ is the population mean, *f_1_* represents the genetic effect, *e_1_* the environmental effect, and *ε_1_* the random error [73].

### 4.3 Statistical analysis

The 153 RILs were classified into three categories following Singh and Rajaram [66,71]: homozygous parental-type susceptible (HPTS), homozygous parental-type resistant (HPTR), and OTHER (responses differing from both parents). The number of resistance genes was estimated using Mendelian segregation analysis [74,75]. A Chi-squared (χ²) test was used to assess goodness-of-fit to expected segregation ratios for two to five independent resistance loci. Correlation for FDS across environments were performed using IBM SPSS Statistics (Version 24) [76]. A one-way ANOVA for FDS across environments was performed using R software (version 4.5.2) [77], with statistical analyses tested with Levene’s test using the car package for variance homogeneity. Following a significant ANOVA, Duncan’s multiple range test (*agricolae* package) was used for post-hoc comparisons at α = 0.05 [78].

### 4.4 Genotyping, genetic mapping, and QTL analysis

Genomic DNA was extracted from the parents and approximately 20 seedlings per RIL using the CTAB method [79]. Genotyping was conducted using the DArT–GBS high-throughput platform (https://www.diversityarrays.com) at CIMMYT headquarter. A total of 84,300 presence-absence variation (PAV) markers and 51,348 SNP markers were obtained. After filtering for distorted segregation (*P* < 0.001), monomorphic loci, and markers with >20% missing data, 5,468 polymorphic markers were retained for linkage map construction. Genetic linkage maps were constructed using JoinMap 4.1 (Van Ooijen 2006) at a LOD threshold of 15.0 [80]. QTL analysis was performed using IciMapping 4.1 [81], based on FDS from each environment, and BLUP values obtained from multi-environmental data. For each locus, LOD scores, phenotypic variance explained (PVE), and additive effects were estimated. Significant QTLs (*P* < 0.05) were identified after 1,000 permutations, and MapChart (Voorrips 2002) was used to visualize linkage maps [82].

### 4.5 Molecular marker analysis

Both parents were genotyped with markers linked to 15 known *Yr* resistance genes (*Yr5/Yr7/YrSP, Yr9, Yr10, Yr15, Yr17, Yr18, Yr26, Yr27, Yr28, Yr29, Yr30*, *Yr36,* and *Yr46*). The primer sequences are in S2 Table. Among these, *csLV46G22* (E. Lagudah, personal communication) for *Yr29*, *KASPYr27* for *Yr27*, and *Xgwm533* for *Yr30* were polymorphic between two parents and subsequently used to genotype the entire RIL population [47].

Conventional PCR amplification protocols for the above genes were validated following Dreisigacker et al [79]. Each 10 μL reaction contained 1 μL genomic DNA (100 ng/μL), 5 μL 2 × PCR Master Mix, 1 μL primer mix (forward and reverse), and 3 μL ddH_2_O. The KASP PCR reactions (5 μL) included 1 μL DNA (100 ng/μL), 2.5 μL 2 × KASP Master Mix, 0.1 μL FAM primer, 0.1 μL HEX primer, 0.2 μL common primer, and 1.1 μL water. The thermocycling profile was: pre-denaturation at 95 °C for 5 min; 35 cycles of 95 °C for 1 min, 58 °C for 20 s, 72 °C for 45 s; followed by final extension at 72 °C for 10 min and hold at 4 °C. PCR products amplified with *csLV46G22* were digested with *BspeI* endonuclease (37 °C for 1 h) and resolved by 1.0% agarose gel electrophoresis. KASP markers were analysed by real-time PCR (RT-PCR).

### 4.6 Expression analysis of candidate genes

In the greenhouse, flag leaves of the adult plants from the parental lines ‘Apav#1’ and ‘Kijil’ were inoculated with a mixture of *Puccinia striiformis* f. sp. *ttritici* races CYR33 and CYR34. Samples were collected at 0 and 48 hours post-inoculation, with three biological replicates per genotype and time point. The collected samples were immediately frozen in liquid nitrogen and stored at –80°C. Total RNA was extracted using TRIzol reagent. Sequencing was performed by Novogene Co., Ltd. (Beijing, China) on an Illumina NovaSeq X Plus platform (USA) with a PE150 configuration, generating >10 Gb clean data per sample. To identify genes actively expressed within the target QTL interval, we analyzed the transcriptome data. Raw read counts were normalized to Fragments Per Kilobase of transcript per Million mapped fragments (FPKM) to generate a standardized expression matrix. The expression levels of all annotated genes within the target interval were extracted for both genotypes and the two time points. Genes with an average expression level FPKM > 1 in at least one time point in either genotype were retained as transcriptionally active. Functional annotation from the Ensembl Plants (https://plants.ensembl.org/index.html) was then integrated to prioritize actively expressed genes potentially linked to disease resistance, resulting in a refined candidate gene list for experimental validation. The raw sequencing data have been deposited in the NCBI SRA under BioProject accession number PRJNA1456677.

Candidate genes identified from the RNA-seq data were subsequently validated by real-time qRT–PCR. Flag leaf samples at the adult plant stage were collected from the resistant parent Kijil and the susceptible parent Apav#1 at 0, 12, 24, and 48 hours post-inoculation with a mixture of *Pst* races CYR33 and CYR34, with three biological replicates per time point. Total RNA was extracted using a plant total RNA extraction kit and reverse-transcribed into cDNA (HiScript Ⅲ 1st Strand cDNA Synthesis Kit). The qRT–PCR was performed on a CFX96 Touch Real-Time PCR Detection system (Bio-Rad) with ChamQ Universal SYBR qPCR Master Mix. The wheat housekeeping gene *TaGAPDH* was used as an internal reference. Gene-specific primers spanning introns were designed (S2 Table).

### 4.7 Analysis of epistatic interactions and effect stability

To analyze the hierarchical interactions between *Yr29*, *Yr30*, and *QYr.hzau-2BS* as well as the stability of their effects, linear model analysis and subgroup analysis were conducted. A linear model incorporating all main effects and pairwise interaction terms was constructed [83]:


$$\begin{array}{c} Y=\beta_{\text{0}}+\beta_{\text{1}}G_{\text{1}}+\beta_{\text{2}}G_{\text{2}}+\beta_{\text{3}}G_{\text{3}}+\beta_{\text{1}\text{2}}G_{\text{1}}G_{\text{2}}+\beta_{\text{1}\text{3}}G_{\text{1}}G_{\text{3}}+\beta_{\text{2}\text{3}}G_{\text{2}}G_{\text{3}}+\epsilon \end{array}$$ (2)

where *Y* represents the phenotypic value for disease severity, and *G*_1_, *G*_2_, and *G*_3_ correspond to the genotype variables of *Yr29* (*csLV46G22*), *Yr30* (*Xgwm533*) and *QYr.hzau-2BS* (KASP_2BS), with resistance alleles coded as 1 and susceptibility alleles coded as 0. This model was used to test the additive effects of each gene (β*_1_*, β*_2_*, β_3_) and the epistatic interaction effects between them (β*_12_*, β*_1_*_3_, β*_2_*_3_). Significant interaction terms (*P* < 0.05) indicate the presence of epistasis, where positive estimates denote antagonistic effects and negative estimates denote synergistic effects.

Furthermore, to evaluate the background dependence of *QYr.hzau-2BS*, the population was subdivided into subgroups based on the genotypes of *Yr29* and *Yr30*, respectively. Within each subgroup, the additive effect (δ) of *QYr.hzau-2BS* was estimated independently using a simple linear regression model [84]:


$$\begin{array}{c} Y=\alpha +\delta G_{\text{3}}+\varepsilon \end{array}$$ (3)

All statistical analyses were performed using R software (version 4.5.2) [77], with model fitting carried out using the lm() function and the significance of effects assessed via *t*-tests (α = 0.05).

## Supporting information

S1 Fig Stripe rust responses of the parents and the susceptible check (SY) at the adult plant (A) and seedling stages (B). (A) Adult plant field phenotypes of the susceptible parent ‘Apav#1’ (left) and the resistant parent ‘Kijil’ (right). (B) Seedling infection types with *Pst* race CYR34. SY represents susceptible check.
(TIF)

S2 Fig Association analysis for *QYr.hzau-2BS* using the KASP marker with stripe disease severity across seven environments. The sample size was adequately designed. “−” indicates the absence of the QTL locus, whereas “+” indicates its presence. Significance levels are denoted by asterisks (^*^, *P* < 0.05; ^**^, *P* < 0.01; ns : non-significant.).
(TIF)

S1 Table Final disease severity of the 153 RILs across seven environments used for QTL analysis. YR2016Y, final stripe rust severity, Ciudad Obregon (Mexico) 2015–2016; YR2016T, Toluca (Mexico) 2016; YR2017T, Toluca (Mexico) 2017; YR2017B, Batan (Mexico) 2017; YR2020EZ, Ezhou (China) 2020; YR2021EZ, Ezhou (China) 2021; YR2022EZ, Ezhou (China) 2022.
(XLSX)

S2 Table Primer sequences for molecular markers and gene expression analysis. (XLSX)

S3 Table Multi-environment t-test analysis for the *QYr.hzau-2BS* locus. The “ + ” and “-” indicate the presence and absence of respective resistance alleles, respectively.
(XLSX)

S4 Table Expression profiles of annotated genes within the *QYr.hzau-2BS* interval. Expression levels (Fragments per kilobase of transcript per million mapped fragments, FPKM) of all annotated genes within the target *QYr.hzau-2BS* physical interval in flag leaf samples of the resistant wheat line ‘Kijil’ and susceptible line ‘APAV#1’ at 0 (K0) and 48 (K48) hours post-inoculation with a mixture of *Puccinia striiformis* f. sp. *tritici* races CYR33 and CYR34. Three biological replicates are shown for each condition. Genes with an average FPKM > 1 across replicates in at least one time point were considered expressed and retained for further analysis, as indicated in the text.
(XLSX)

S5 Table Phenotypic data of different groups across seven environments used for analysis. The “ + ” and “-” indicate the presence and absence of respective resistance alleles, respectively.
(XLSX)

S6 Table Effects of *QYr.hzau-2BS* on stripe rust severity in different genetic backgrounds of *Yr29* and *Yr30.* Data from subgroup analyses within the RIL population. BLUPM and BLUPC are the best linear unbiased predictions for Mexican and Chinese environments, respectively. ^a^ + , Presence; -, Absent. ^b^ The additive effect represents the estimated change in disease severity (%) attributed to the resistance allele of *QYr.hzau-2BS* within each subgroup. A negative value indicates that the allele reduces disease severity (enhances resistance). ^c^ P values were derived from simple linear regression within each subgroup. ^d^ Significance levels: ***, *P* < 0.001; **, *P* < 0.01; *, *P* < 0.05; ns, not significant.
(XLSX)

S1 File Integrated dataset for QTL mapping and candidate gene validation. The file contains the data used for genetic map construction, QTL mapping, and association analysis in this study.
(XLSX)
